# Is There an Association between Traumatic Dental Injury and Social Capital, Binge Drinking and Socioeconomic Indicators among Schoolchildren?

**DOI:** 10.1371/journal.pone.0118484

**Published:** 2015-02-26

**Authors:** Haroldo Neves de Paiva, Paula Cristina Pelli Paiva, Carlos José de Paula Silva, Joel Alves Lamounier, Efigênia Ferreira e Ferreira, Raquel Conceição Ferreira, Ichiro Kawachi, Patrícia Maria Zarzar

**Affiliations:** 1 Department of Dentistry, Federal University of Jequitinhonha and Mucuri Valleys, 39100-000, Diamantina, Brazil; 2 Department of Child and Adolescent Health, Federal University of Minas Gerais, 30130-100, Belo Horizonte, Brazil; 3 Department of Dentistry, Federal University of Jequitinhonha and Mucuri Valleys, 39100-000, Diamantina, Brazil; 4 Department of Public Oral Health, School of Dentistry, Federal University of Minas Gerais, 31270-901, Belo Horizonte, Brazil; 5 Department of Social and Behavioral Sciences, Harvard School of Public Health and Medical School, 617495.1000, Harvard, Boston, MA, United States of America; 6 Pediatric Dentistry and Orthodontics, School of Dentistry, Federal University of Minas Gerais, 31270-901, Belo Horizonte, Brazil; Örebro University, SWEDEN

## Abstract

**Objectives:**

Traumatic dental injury is defined as trauma caused by forces on a tooth with variable extent and severity. The aim of the present study was to investigate the prevalence of traumatic dental injury and its association with overjet, lip protection, sex, socioeconomic status, social capital and binge drinking among 12-year-old students.

**Research Design and Method:**

A cross-sectional study was conducted with a sample of 633 12-year-old students. Data were collected through a clinical exam and self-administered questionnaires. Socioeconomic status was determined based on mother’s schooling and household income. The Social Capital Questionnaire for Adolescent Students and Alcohol Use Disorders Identification Test (AUDIT-C) were used to measure social capital and binge drinking, respectively.

**Results:**

The prevalence of traumatic dental injury was 29.9% (176/588). Traumatic dental injury was more prevalent among male adolescents (*p* = 0.010), those with overjet greater than 5 mm (*p* < 0.001) and those with inadequate lip protection (p < 0.001). In the multiple logistic regression analysis, overjet [OR = 3.80 (95% CI: 2.235–6.466), p < 0.0001], inadequate lip protection [OR = 5.585 (95% CI: 3.654–8.535), p < 0.0001] and binge drinking [OR = 1.93 (95% CI: 1.21–3.06), p = 0.005] remained significantly associated with traumatic dental injury.

**Conclusions:**

The present findings suggest that a high level of total social capital and trust are not associated with TDI in adolescents, unlike binge drinking. The effects of social and behavioral factors on TDI are not well elucidated. Therefore, further research involving other populations and a longitudinal design is recommended.

## Introduction

Traumatic dental injury (TDI) is one of the most serious public health problems affecting children and adolescents due to the high prevalence rates, psychosocial impact and treatment costs [1, 2]. TDI has become one of the most important oral health problems since the reduction in the prevalence and severity of dental caries [3].

Population-based studies addressing the permanent dentition report an approximately 20% prevalence rate of TDI among children and adolescents [4], with rates ranging from 6% [5] to 58.6% [6] among 12-year-olds. The etiology and characteristics of TDI as well as predisposing factors, such as sex, accentuated overjet, inadequate lip protection and socioeconomic status, have been widely studied [2, 4, 7, 8]. Social and behavioral factors have also been associated with maxillofacial and dental trauma[9, 10], such as hazardous alcohol intake [11] and social capital [12, 13].

Biological factors, such as accentuated overjet and inadequate lip protection, can predispose individuals to TDI [14, 15]. Overjet is the overlap of the maxillary incisors in relation to the mandibular incisors on the horizontal plane and increases in function of anteroposterior relationships of the maxillae and mandible as well as the type of facial growth. The risk of TDI increases proportionally to the increase in overjet [14]. Adequate lip protection is classified when the maxillary incisors are completely covered by the upper lip when the jaw is at rest. The lip absorbs impact, thereby protecting the teeth during a collision. Thus, individuals with inadequate lip protection are more prone to fracturing their anterior teeth [6, 11, 16].

Based on the biopsychosocial model, healthcare professionals should not only consider signs and symptoms, but should be concerned with biological, psychological and social factors as determinants of health and illness [17]. Social capital regards the characteristics of social organization that enhance the efficacy and efficiency of society, such as trust and relationship networks [18]. The concept of social capital has been used in a vast array of disciplines and a growing number of studies have suggested that adequate social capital is beneficial to health [12, 19–22]. Indeed, social capital is increasingly studied for its contextual influence on health, with emphasis given to the characteristics of the social environment, in contrast to past studies in which the focus was merely on the individual. Researchers in public health have sought explanations in social capital for the heterogeneity of health status across geographic areas and different social contexts, emphasizing that relationships exert an important impact on health and wellbeing [23]. Thus, health status can be measured based on social structure and not merely on individual determinants [21, 22]. Social capital may be considered a determinant of the health of a population, as health is influenced by demographic, socioeconomic and behavioral factors as well as the ability to cope with problems [24].

A few studies have revealed the relationship between social social capital on oral health among young people. The association between these aspects is not uniform. A higher degree of trust has been associated with better oral health, whereas a higher degree of informal control in the community has been associated with worse oral health in a sample of college students aged 18 and 19 years [25]. Associations between neighborhood/individual social capital and oral health-related quality of life have also been assessed in pregnant and postpartum women. One study found that individuals living in neighborhoods with high social capital were less likely to report the occurrence of toothache [26]. Despite the increase in number of studies on oral health-related social capital, few investigations have addressed the association between social capital and TDI among adolescents [12, 13].

To date, only two investigations have studied associations between social capital and TDI among adolescents [12, 13]. The results of a study developed by Patussi et al. [12] revealed that adolescents with a lower prevalence rate of TDI had a greater chance of having a high level of social capital. However, a study developed by Moysés et al. [13] involving a sample of 2200 12-year-old students in the city of Curitiba (southern Brazil) found no association between social capital (social cohesion) and dental trauma.

A number of studies have reported an association between binge drinking (five or more alcoholic drinks on a single occasion) [27] and TDI in adolescents [11, 28]. It is important to include this variable in studies that investigate associations between TDI and social/behavioral determinants. A set of factors may be associated with binge drinking among adolescents, such as the need for socialization, peer expectations and beliefs as well as family and social contexts [29]. The consumption of alcoholic beverages reduces self-control and increases the risk of anti-social behavior, crime, poor academic performance, interpersonal violence and accidental injuries, which could culminate in maxillofacial trauma [9] and TDI [11, 28] Although the literature offers studies that have investigated the association between craniofacial fractures and the consumption of alcoholic beverages [9, 30–32], the few studies that have addressed TDI have been conducted in a hospital setting with young adults [33, 34]. However, a significant association has been found between the use of alcoholic beverages by adolescents aged 14 to 19 years and TDI, independently of the other variables analyzed [11].

The development of epidemiological studies that investigate the relationship between social capital and both TDI and associated factors, such as binge drinking, is important to ensuring that affected individuals receive assistance according to social context and determinants. Moreover, the findings of such studies can contribute to the implantation of educational programs at schools and in communities directed at adolescents at risk.

Despite the relevance of social capital to health, the few studies that have investigated the relationship between social capital and oral health report conflicting results. Moreover, the majority did not employ a validated instrument developed specifically for administration to adolescent samples.

Therefore, the aim of the present study was to investigate the prevalence of TDI and its association with overjet, lip protection, sex, socioeconomic status, social capital and binge drinking among 12-year-old students in a medium-size city in Brazil.

## Methods

### Study design and sample

The present cross-sectional study was carried out in southeastern Brazil in a municipality with 46,372 inhabitants, an 83.4% literacy rate, a human development index (HDI) of 0.748 and an income HDI of 0.752 between February and April 2013. A total of 7,474 schoolchildren are enrolled in elementary schools in urban and rural areas in the municipality (477 in private schools and 6,997 in public schools) [35]. The study population included all 633 12-year-old students enrolled at all 13 public and private schools in urban areas. The following estimates were obtained for the sample: the prevalence of trauma in exposed and non-exposed groups, prevalence ratios (PR), 95% confidence level and an 80% test power.

Training was carried out with color slides of each type of injury in the permanent dentition, with two images of each injury. The calibration exercise was then performed in a pilot survey through clinical examinations of 12-year-old students who did not participate in the main study. Intra-examiner and inter-examiner agreement was determined using the Kappa index. The intervals between both examinations were 15 days. The methods were first tested in a pilot study involving a convenience sample of 101 students who were not part of the main study. The results of the pilot study revealed no need for changes to the proposed methodology. The team consisted of an examiner who had undergone a training and calibration exercise (intra-examiner Kappa = 0.79; inter-examiner Kappa [compared to a researcher/dentist who is an expert in dental trauma] = 0.85) and annotator.

### Collection of clinical data

Data collection was performed at the schools at a previously scheduled day and time. For the clinical exam, the student was seated in front of the examiner. TDI was recorded based on the classification proposed by Andreasen et al. [16]. All permanent incisors were examined using a sterilized dental instrument with illumination provided by a head lamp (Petzl Zoom head lamp; Petzl America, Clearfield, UT, USA). The teeth were cleaned and dried with gauze. Each dental crown was examined for the loss of tooth structure, discoloration, avulsion or the presence of restoration with the aid of a mouth mirror and compared to the contralateral crown.

A wooden tongue depressor with a straight tip was used to measure overjet. For such, the teeth were positioned in centric occlusion and overjet was measured from the vestibular face of the mandibular incisor to the incisal face of the most prominent maxillary incisor and marked with graphite on the tongue depressor. The measurement was then made using digital calipers. Accentuated overjet was determined as greater than 5 mm.

Lip coverage was evaluated based on the method proposed by O’Mullane [14] and was considered adequate when the lip at rest covered the maxillary incisors.

### Collection of non-clinical data

Social capital was investigated using the Social Capital Questionnaire for Adolescent Students, which was developed and validated by our research team. This questionnaire is composed of items selected from the national and international literature and has been submitted to face validation, content analysis and analyses of internal consistency, reliability and reproducibility. The factor analysis grouped the 12 items into four subscales: Social Cohesion at School; Network of Friends at School; Social Cohesion in the Community/Neighborhood; and Trust at School and in the Community/Neighborhood. Social capital scores range from 12 to 36 points, with a higher score denoting greater social capital [36].

The Alcohol Use Disorders Identification Test (AUDIT) was used to identify the consumption of alcoholic beverages. This fast, easy-to-administer test has been validated for use on Brazilian populations [37] and is considered adequate for adolescents [11, 38, 39]. The short version (AUDIT C) has three items addressing the frequency and quantity of alcohol intake [40] and has also been validated for use in Brazil [41]. The third item was used to classify binge drinking [27], which was dichotomized as 0 (never consumed five or more alcoholic beverages on a single occasion) and 1 (consumed five or more alcoholic beverages on a single occasion at a frequency of once a month to daily).

The socioeconomic indicators employed were monthly household income and mother’s schooling. Household income was determined based on the sum of all salaries received by economically active residents in the home and categorized based on the current Brazilian minimum salary; the threshold was the median response. Mother’s schooling was defined as the number of years of study, with seven years used as the cut-off point; the threshold was the median response. According to the Brazilian Institute of Geography and Statistics [35], the mean years of study of the Brazilian population is 7.4 years. Thus, the cutoff point of 7 years is related to the beginning of middle school. These socioeconomic variables were collected using a form completed by parents/guardians along with a signed letter of informed consent.

The clinical charts and questionnaires were coded to allow the correlation of the findings while ensuring confidentiality (no participant was identified by name). The questionnaires were self-administered in the classroom without the presence of the teacher.

### Statistical analysis

Data analysis was performed using the Statistical Package for the Social Sciences (SPSS for Windows, version 19.0, SPSS Inc, Chicago, IL, USA) and included frequency distribution and association tests. The chi-square test was used to determine the statistical significance of associations between TDI and the independent variables (*p* < 0.05). Univariate logistic regression analysis was used for the total capital social score and trust subscale score. Non-significant variables were discarded. Variables with a *p*-value < 0.20 in the univariate analysis were incorporated into the multivariate logistic regression analysis, the aim of which was to correlate statistically significant variables.

### Ethical considerations

This study received approval from the Human Research Ethics Committee of the Federal University of Minas Gerais (Brazil) (COEP-317/11). All parents/guardians signed a statement of informed consent authorizing the participation of their children. All adolescents also signed a statement of informed consent.

## Results

The sample consisted of 588 students (participation rate: 92.89%). The male sex accounted for 48.7% (n = 286). The reasons for dropouts were non-authorization from parents/guardians or adolescents (4.62%; n = 28) and failure to complete the questionnaires (2.9%; n = 17).

The vast majority (92.2%; n = 542) was enrolled at public schools. A total of 75.2% (n = 442) of adolescents were from families that earned up to three times the Brazilian monthly minimum wage and 63.9% (n = 376) of the mothers had more than seven years of schooling. No significant associations were found between TDI and social economic indicators (Table 1).

**Table 1:** Distribution of 588 12-year-old students according to traumatic dental injury and independent variables, Brazil, 2014.

	Traumatic dental injury		
Independent variables	Present	Absent	*p-value* *
**Sex**	**(n) (%)**	**(n) (%)**	
Male	100 (34.9)	186 (65.1)	0.010*
Female	76 (25.1)	226 (74.9)	
**Overjet**			
≤ 5 mm	120 (23.9)	382 (76.1)	<0.0001*
> 5 mm	56 (65.1)	30 (34.9)	
**Lip protection**			
Adequate	41 (12.8)	276 (87.2)	<0.0001*
Inadequate	135 (49.9)	136 (50.1)	
**Monthly household income**			
≤ 3 times minimum salary	136 (30.6)	307 (69.4)	0.665*
> 3 times minimum salary	41 (28.2)	104 (71.7)	
**Mother’s schooling**			
0 to 7 years	69 (32.8)	141 (67.2)	0.307*
8 year or more	106 (28.1)	270 (71.8)	
**Trust**			
High	11 (23.9)	35 (76.1)	0.014*
Low	166 (29.7)	393 (70.3)	
**Binge drinking**			
Never	121(26.7)	331(73.2)	0.002*
Less than once a month to daily	55(59.5)	81(59.5)	

*chi-square test.

Falls constituted the main etiological factor for the occurrence of TDI (42.7%; n = 38). TDI occurred most often on the street (34.8%; n = 31) and in the afternoon (59.3%; n = 48) more than a year prior to the study (55.2%; n = 32). Only 27.3% of the students with TDI received some type of treatment, the most frequent of which was a composite resin restoration (17.1%; n = 22).

Two hundred nineteen fractured teeth were identified in 176 adolescents, resulting in a 29.9% prevalence rate of TDI. The prevalence was significantly higher in the male sex (34.9%) than the female sex (25.1%) (*p* < 0.010) (Table 1). A total of 49.43% (n = 87) of the 176 adolescents with TDI required restorative crown treatment.

Binge drinking was considered a confounding variable in the logistic regression model, as risk behavior stemming from binge drinking is a possible mediator of TDI. In the univariate analysis, significant associations were found between TDI and both the trust subscale score [crude OR = 0.867 (95% CI: 0.773 to 0.971), p = 0. 014] and total social capital score [crude OR = 0.955 (95% CI: 0.914 to 0.998), p = 0. 039] (Table 2).

**Table 2:** Results of univariate logistic regression in exploratory analysis of social capital among 588 12-year-old students, Brazil, 2014.

Variable		OR (95% CI) Crude	*p* *	OR (95% CI) Adjusted	*p* *
**Social cohesion** *	High	1.00			
	No	0.927(0.837–1.027)	0.149		
**Network of friends** *	High	1.00			
	Low	1.009(0.897–1.135)	0.881		
**Cohesion of friends** *	High	1.00			
	Low	0.896(0.791–1.015)	0.083		
**Trust** **	High	1.00		1.00	
	Low	0.867(0.773–0.971)	0.014	0.920 (0.808–1.048)	0.210
**Total social capital** **	High	1.00		1.00	
	Low	0.955(0.914–0.998)	0.039	0.9863(0.934–1.035)	0.516

* Not incorporated into multiple regression analysis due to *p* > 0.20 in univariate logistic regression analysis.

** Removed from final model due to *p* < 0.20.

However, both independent variables lost statistical significance in the multivariate logistic regression model [OR = 0.920 (95% CI: 0.808 to 1.048), p = 0. 210; and OR = 0.986 (95% CI: 0.934 to 1.035), p = 0.516, respectively] (Table 3).

**Table 3:** Multivariate logistic regression analysis of dental trauma and independent variables among 588 12-year-old schoolchildren, Brazil, 2014.

Dependent variable	Independent variables		OR (95% CI) crude	*p* *	OR (95% CI) adjusted	*p* *
**TDI**						
	**Overjet**	≤ 5mm	1.00		1.00	
		> 5mm	5.942(3.646–9.686)	<0.0001	3.802(2.235–6.466)	<0.0001
	**Lip coverage**	Adequate	1.00		1.00	
		Inadequate	6.682(4.455–10.022)	< 0.0001	5.585(3.654–8.535)	<0.0001
	**Binge drinking**	No	1.00		1.00	
		Yes	1.857*1.244–2.773)	0.002	1.928(1.213–3.063)	0.005
	**Sex**	Female	1.00		1.00	
		Male	1.599(1.120–2.282)	0.010	1.334(0.889–2.000)	0.164
	**Trust**	High	1.00		1.00	
		Low	0.867(0.773–0.971)	0.014	0.924(0.811–1.053)	0.236

OR, Odds ratio; CI, confidence interval.

* Ajusted for sex.

Overjet, lip protection, sex, binge drinking and trust were incorporated into the multiple logistic regression model. Accentuated overjet [OR = 3.80 (95% CI: 2.235–6.466), p < 0.0001], inadequate lip protection [OR = 5.585 (95% CI: 3.654–8.535), p < 0.0001] and binge drinking [OR = 1.93 (95% CI: 1.21–3.06), p = 0.005] remained significantly associated with traumatic dental injury (Table 3).

## Discussion

The recognition that one’s social context can exert an influence on health-related behavior highlights the particular importance of social capital in contemporary epidemiology [42]. However, the vast majority of the growing number of studies on this issue involve samples of adults [12] and little is known regarding the relationship between social capital and health outcomes among adolescents, beyond the use of one or two questions addressing one’s network of friends and neighborhood cohesion [43].

Associations between TDI and social capital, the physical environment and public policies were evaluated in a sample of 2200 12-year-old students in the city of Curitiba (southern Brazil). Social capital was investigated by social cohesion, which was not found to be significantly associated with TDI, which is in agreement with the present findings. However, the physical environment and public policies were important prediction factors for health. The authors stress that conclusions regarding the probable role of social capital in the health of the population should be interpreted with caution due to the considerable variation in the measures used to evaluate social cohesion [13]. The method employed to investigate the association between TDI and social capital in the present study makes this investigation unique, as an assessment tool developed and validated specifically for adolescent students was used for this purpose.

The 29.9% prevalence rate of TDI among the 12-year-old students analyzed is higher than rates reported in most Brazilian studies [44–46], but lower than rates reported for cities located in the southern region of the country [6, 11, 47, 48]. The massive size of Brazil results in huge cultural, social and economic differences, which hinders the establishment of a general pattern regarding TDI [40]. Lower prevalence rates of TDI among 12-year-olds have been reported in Spain [5], India [50], Nigeria [51, 52], Ireland [15] and Jordan [53].

The most prevalent types of TDI were enamel fractures, followed by enamel/dentin fractures. These constitute injuries of lower magnitude subject to interventions of low complexity available at public healthcare services. Similar data have been reported for other parts of Brazil, which may signify negligence in the treatment of TDI as well as restricted access to dental services for the majority of the population [45]. The low level of knowledge on TDI on the part of the population, especially injuries of low intensity and a low degree of morphological impairment, may lead individuals to fail to seek the required care, which may be an additional factor explaining the low percentage of treated TDI.

The male sex was more affected by TDI and most incidents occurred in the street. There is a consensus in the literature that boys are at greater risk for TDI in adolescence [11, 38, 54, 55] due to the tendency to participate more in sports and outdoor activities [2, 46]. Moreover, TDI was significantly more prevalent among individuals with overjet ≥ 5 mm (p < 0.0001) and inadequate lip protection (p < 0.0001), which is in agreement with most studies that investigate predisposing clinical factors [49].

Household income and mother’s schooling were used as socioeconomic indicators, but neither was significantly associated with the occurrence of the outcome. Indeed, the literature reports divergent findings regarding the association between TDI and socioeconomic status [7]. Feldens et al. [56] found that socioeconomic status exerted an influence in different contexts, highlighting the importance of cultural aspects, social capital and social vulnerability. The conflicting results may partially be explained by the different indicators and cutoff points employed.

Alcohol is the most consumed psychoactive substance among young people, the main consequences of which are physical, social and psychological problems [57]. Alcohol intake is considered a risk factor for antisocial behavior, crime, poor academic performance, interpersonal violence, accidental injuries and traffic accidents, which can culminate in maxillofacial and dental trauma [9, 11]. An association between hazardous drinking and TDI has been reported among students aged 14 to 19 years [11], which is in agreement with the present findings. However, it should be stressed that binge drinking acted as a confounding variable, influencing the results of the multivariate logistic regression analysis and revealing that social capital was not significantly associated with TDI.

A number of studies have related social capital to general health [21, 58]. However, few studies have revealed the impact of social capital on oral health [20, 25] and specifically TDI [12] among young people. The association between social capital and self-rated oral health is not uniform [20, 26]. The literature demonstrates that trust and reciprocity emerge through interactions and mutual assistance on the collective level and social capital is therefore measured based on social structure. However, neither the total social capital score nor the trust subscale score remained significantly associated with TDI in the present investigation. These findings are in disagreement with data reported by Patussi et al. [12], who found a lower prevalence rate of TDI in neighborhoods with higher social capital, which offered better environmental contexts and larger social networks, perhaps leading to a lower likelihood of determinant factors of TDI [12]. The social and cultural context may partially explain the lack of an association between TDI and social capital, as no significant associations were found between the outcome and social determinants either.

The loss of statistical significance in the multivariate logistic regression analysis may be partially explained by the nature of the injuries. It is possible the enamel fractures and small enamel/dentin fractures were overlooked by the adolescents and their parents/caregivers. Ramos-Jorge et al. [59] developed a study to evaluate the recognition of TDI on the part of parents/caregivers as well as the impact of TDI on activities of daily living and the quality of life of children. The authors found that many parents/caregivers did not recognize the occurrence of TDI in their children, the majority of which were cases of enamel fracture. Thus, the impact of oral health on quality of life was directly associated with the recognition of the occurrence of TDI. These findings lend support to the hypothesis that social capital is affected by the failure to recognize TDI as an adverse health condition, since the majority of TDIs in the present study were also enamel fractures, which have less social, esthetic and functional impact. To be recognized as an adverse oral health condition with an impact on quality of life, it is necessary for individuals to be aware of the presence of TDI, its consequences and the need to seek treatment. Thus, the low prevalence rate of severe TDI, the low rate of restorative treatment needs and the smaller effect on wellbeing may explain the absence of a significant association with social capital.

The use of alcoholic beverages by adolescents stimulates risk behavior that can lead to injuries. It is therefore necessary to develop studies at urgent care reference centers that investigate the association between binge drinking and TDI in adolescents. Such studies would enable investigations into the influence of social capital on these aspects.

The present study has limitations that should be considered. Although confidentiality of the information was ensured, some data may have been underestimated due to embarrassment or fear of answering affirmatively to questions regarding binge drinking. Moreover, the cross-sectional study design does not allow the determination of causality. The main focus of the purpose of the study and the methodology proposed was to investigate TDI and associated factors in adolescent students. Nonetheless, one should bear in mind that a percentage, however small, of adolescents involved in risk behaviors for binge drinking and TDI may have dropped out of the educational system, which is another limitation of the study. The current Brazilian socioeconomic policy encourages students to remain in school and even employs financial stimulation through the School Grant Program, the aim of which is to combat poverty and social inclusion through access to education by offering eligible families a monthly grant to be invested in the education of their children to avoid obligating children to work at an early age to contribute to the household income. This policy has resulted in a substantial reduction in truancy [60].

## Conclusions

For many years, dentistry has emphasized individual clinical factors as predisposing aspects with regard to oral conditions. The present findings suggest that a high level of total social capital and trust are not associated with TDI in adolescents, unlike binge drinking. The effects of social and behavioral factors on TDI are not well elucidated. To a certain extent, the present results confirm the lack of statistically significant association between TDI and social capital. However, further studies are needed to gain a better understanding the role of contexts that shape such behaviors. Therefore, further research involving other populations and a longitudinal design is recommended.

### Ethical approval

This study obtained approval from the Research Ethics Committee of the Federal University of Minas Gerais (COEP-317/11).
